# Biomechanical evaluation of peak reverse torque (PRT) in a dynamic compression plate-screw construct used in a goat tibia segmental defect model

**DOI:** 10.1186/s12917-019-2058-7

**Published:** 2019-09-05

**Authors:** Remigiusz M. Grzeskowiak, Carrie Wheeler, Elizabeth Taylor, James Lillich, James Roush, Alexandru S. Biris, David E. Anderson

**Affiliations:** 1 Large Animal Clinical Sciences, University of Tennessee College of Veterinary Medicine, |2407 River Dr, Knoxville, TN 37996 USA; 2 Kansas State University College of Veterinary Medicine, |1700 Denison Ave, Manhattan, KS 66506 USA; 3 The University of Arkansas at Little Rock, Center for Integrative Nanotechnology Sciences, |2801 S. University Avenue, Little Rock, AR 72204 USA

**Keywords:** DCP, Orthopedic plate, Screws, Reverse torque, PRT, Animal model, Segmental defect, Osseointegration, Biomechanics, Fracture

## Abstract

**Background:**

Peak reverse torque (PRT) is a valid method to evaluate implants’ secondary stability in the healing bone. The secondary stability is achieved by the implant over time and it has been positively correlated with the implants’ osseointegration level. In other words, peak reverse torque is the force required to break the bone-implant interface. The purpose of this study was to compare the peak reverse torque for the self-tapping and non-self-tapping screws used in a dynamic compression plate–screw–bone construct after 60 days of loading when used to stabilize 2.5-cm defects in the tibia of goats. The second objective was to compare the peak removal torque of the screws placed in the different positions to evaluate the impact of construct biomechanics on implants osseointegration.

**Results:**

In total, 176 non-self-tapping screws and 66 self-tapping screws were used to fix the 8-holes dynamic compression plates to the bones. The screws were placed in the tibiae from proximal (position sites 1,2, 3) to distal (position sites 4,5,6) and were removed 60 days post-implantation. The animals remained weight-bearing throughout the study period. The screws placed in the proximal diaphysis had significantly less peak reverse torque than screws placed in the distal diaphysis in both groups (*p* < 0.05). The peak reverse torque resistance was also significantly less for the non-self-tapping screws as compared with the self-tapping screws (*p* < 0.05). The intracortical fractures in the trans-cortex occurred significantly more frequently during the placement of non-self-tapping screws (*p* < 0.05) as compared with self-tapping screws (*p* < 0.05).

**Conclusions:**

Based on these results, we concluded that self-tapping screws may be expected to maintain a more stable bone-implant interface during the first 60 days of loading as compared with non-self-tapping screws. This should be a consideration for orthopedic surgeons and scientists using bone plates to stabilize non-load sharing fractures when a stable plate-screw-bone interface is needed to ensure prolonged stability.

## Background

Maintenance of the interface between screws and bone is important to ensure adequate stabilization of fractures and to maintain mechanical support for the healing tissue [1, 2]. The screw is a critical linkage to secure bone plates to bone. Assuming that, the plate is sufficiently stiff and resilient under cyclical loading conditions, and then the integrity of the screw-bone interface determines the overall stability of the construct. The bone-screw interface is defined by its primary and secondary stability. Primary stability is obtained by the screw immediately after placing it into the bone and has been associated with several factors: surgical technique, implant design, surface properties, loading, and quality of the bone [1, 3–6]. Secondary stability refers to the long-term stability of the screw-bone interface and is directly related to the osseointegration between the bone and the implant’s surface [3, 7]. Several factors have been described to be of importance in this process: biocompatibility, surface texture, surgical technique, the status of the host tissue, and loading conditions [3, 7]. Secondary stability can be measured using resonance frequency (RF) or peak reverse torque (PRT) [3, 8]. Several studies, mostly on orthodontic implants, have used PRT [3–7, 9–15] showing that peak reverse torque has been positively correlated with the osseointegration process [3, 5, 6, 13–16] and bone density [3, 6, 11, 14].

Various fixation techniques have been described and used to stabilize tibia defects using large animal models [17–21]. These techniques include a single dynamic compression plate fixation [17–19], locking intramedullary nail [20], and double plate fixation [21] resulting in the different mechanical environments for the regenerating bone. The studies that have used a single DCP concluded that this fixation technique provides adequate stabilization for most large animal tibia defect models [17–19].

Dynamic Compression Plate (DCP) is a type of conventional plate commonly used in the fracture repairs [22]. The plate mechanics rely on a transfer of the axial loading forces from the bone to the proximal screws, which transfer the load into the plate; this load is then transferred from the plate back to the distal bone segment via the distal screws. Ground reaction forces are controlled in the same manner but in a reverse direction. The resulting shear (frictional) forces across the plate-bone interface concentrate stress at the plate-screw-bone unit [2, 22]. The plate-screw-bone unit exerts shear forces along the bone-screw thread interface as a result of the torque applied to the screws during insertion when fixing the plate to the bone (approximately 3–5 Nm for 3.5 mm cortical screws placed into human femur) [23, 24]. The mechanical stability of the plate is affected by how well it is fitted against the surface of the bone [2]. With the use of DCP, as the screw is being tightened, the screw head slides down on the decline slope within the screw hole, converting the descending movement of the screw into a gliding movement of the plate [2]. Therefore, during the implant placement, the screw torque generates relative compressional strain on the bone surface and tension in the cortical bone around the screw threads [2]. Each screw in this construct is loaded individually at the screw-bone interface and the farthest screws at each end of the plate tend to experience the largest interface loads [25].

Although the entire construct can be tested via compression, bending, and torsion of the plate-screw-bone construct, those tests do not assess individually the integrity of each screw-bone interface. Peak reversal torque is a valid method to evaluate the implants interface as an indicator of osseointegration. Osseointegration has been positively correlated with the loading conditions around the implant. The axial strength of the plate may be predicated on the axial strength of the weakest screw in the plate-screw-bone construct because this weakening results in transference of loading forces to adjacent screws. The evaluation of each screws’ osseointegration provides insight into this aspect of the plate-screw-bone construct stability. Although the PRT of the various screws has been studied, to our knowledge, studies on reverse torques of screws used in plate-screw-bone constructs after periods of loading are lacking.

The objectives of this study were to measure the peak reverse torque (PRT) of each screw used in a plate-screw-bone construct at the time of its removal after 60 days of in-vivo loading in a non-load sharing, 2.5 cm segmental defect in goats. We hypothesized that the PRT would vary among the screw positions as a result of the cyclical loading construct biomechanics. Secondly, we hypothesized that the ST screws used to fix the plate would have superior PRT compared with that of NST screws after 60 days of cyclical loading.

## Results

All goats remained weight-bearing throughout the study period. A total of 318 screws were used for the study, of which the PRT data for 76 screws were not included in the PRT study due to the following factors: large callous formation around the plate and screw heads (3 plates), plate bending (4 plates), goat removal from the study prior to 60 days (3 plates) and device reading errors (16 screws). The plate bending observed in 4 constructs occurred in animals which showed subjectively evaluated higher level of activity as compared to the other animals. There was no relationship between the weight of the animal and bending of the construct. The remaining 3 animals were removed from the study approximately 1 month after the procedure due to the pullout and displacement of the three most proximal screws resulting in the plate displacement more than 1 cm away from the tibia. The peak reverse torques of 242 screws were included in this study, of which 176 were non-self-tapping (NST) screws and 66 were self-tapping (ST) screws (Table 1).

**Table 1:** Peak Reverse Torque categories for non-self-tapping (NST) and self-tapping (ST) screws: Maximal, High, Medium and Low

Peak Reverse Torque Groups										
NST Screws (Number)						ST Screws (Number)				
Prox to Dist	Max	High	Med	Low	Total	Max	High	Med	Low	Total
1	5	8	13	4	30	3	3	3	2	11
2	5	13	9	2	29	4	4	3	0	11
3	4	2	14	9	29	5	4	2	0	11
4	9	12	8	0	29	9	1	0	1	11
5	16	8	5	0	29	9	2	0	0	11
6	9	11	9	1	30	8	3	0	0	11
Total	48	54	58	16	176	38	17	8	3	66
% of total	27%	31%	33%	9%	100%	57%	26%	12%	5%	100%

Most of the NST screws PRT were categorized as High and Medium, whereas the ST screws PRT were mostly categorized as Maximal and High. Overall screws in the position 1–3 were categorized as Medium and Low, whereas the distal screws in positions 4–6 in the majority were categorized as Maximal and High in both screw types (ST and NST). The last line of the table presents the percentage of overall screws placed in the different categories

Based on evaluation of the initial results of the PRT measurements, PRT data was categorized into four reverse torque ranges: low (t = 0 Nm), medium (0 Nm < t < 0.66 Nm), high (0.66 Nm < t < 2.60 Nm) and maximal PRT (t > 2.60 Nm). After 60 days of loading, 9.09% of all NST screws, as well as 4.55% of all ST screws, were placed in the low PRT category (t = 0 Nm). The 38 ST screws and 44 NST screws (58% of all ST screws and 25% of all NST screws respectively) exceeded 22.6 Nm, the maximum range of the torque driver.

The two-sided Fisher’s Exact Test revealed that the transcortical diaphyseal tibial fractures occurred significantly more frequently in the NST screws group (*p* < 0.05). The fractures however did not influence the PRT after 60 days (*p* > 0.05). During the placement of the screws, the transcortical diaphyseal tibial fractures occurred in 37 NST screws and in 5 ST screws (21% of all NST screws and 8% of all ST screws, respectively). The transcortical fractures in the NST screws were most often observed in the screw position no. 4 and no. 5 (41.4 and 31.03% of all transcortical fractures in NST screws group, respectively) (Table 2). The pattern was not observed in the ST screws where the fractures were equally distributed between each position, from 1 through 5 (20% of all fractures in each position in ST screw group) (Table 2).

**Table 2:** The prevalence of cortical fractures within each screw type for each screw position

	Screw Type					
Position	NST			ST		
	Total (n)	Intracortical Fracture (n)	Intracortical Fracture (% of total)	Total (n)	Intracortical Fracture (n)	Intracortical Fracture (% of total)
1	30	2	6.7	11	1	9.1
2	29	6	20.7	11	1	9.1
3	29	5	17.2	11	1	9.1
4	29	12	41.4	11	1	9.1
5	29	9	31.0	11	1	9.1
6	30	3	10.0	11	0	0

The intracortical fractures occurred in the trans cortex more frequently during the placement of the non-self-tapping screws [36] as compared with the self-tapping screws [5]. The position most commonly associated with the fractures were position no. 4 and position no. 5 in the NST screws group. In the ST screws group the fractures were more equally distributed between the positions

Statistical analysis revealed significant differences between the NST screw PRT and the ST screw PRT (*p* < 0.05). NST screws were significantly more likely to result in PRT less than 0.66 Nm (Table 1). ST screws were significantly more likely to have PRT greater than 0.66 Nm (Table 1). Significant differences in PRT were also found based on the screw insertion position. Screws placed in the proximal tibia (positions 1, 2, and 3) had significantly lower PRT as compared with those placed in the distal tibiae (position 4, 5, and 6) (Table 1). The relationship of screw position and PRT was similar among ST and NST screws (Table 1).

## Discussion

To our knowledge, measurement of PRT has not been reported after a sustained period of loading in vivo. The model used herein is a non-load sharing model resulting in significant cyclical forces being applied to the bone-screw-plate construct and especially at the bone-screw interface. Similar to previous studies, the DCP provided adequate fixation with satisfactory stability for the non-load sharing tibia defect during this 60-day period of study [17–19].

Screws placed proximal to the ostectomy tended to exhibit lower PRT than the screws placed distal to it. Lower torsional forces needed to break the bone-implant interface have been related to less implant osseointegration [3, 5, 6, 13–16]. There are several factors which are of importance in the osseointegration process: biocompatibility, surface quality, surgical technique, the status of the host tissue, and loading conditions [3, 7]. In the DCP-screw-bone construct, the screws on each end of the plate tend to be exposed to higher loads [2, 22] and this has been negatively associated with implant osseointegration [3, 7]. Bottland et al. showed that screws placed remotely to the fracture or osteotomy sustain greater loads than the screws adjacent to the fracture [26]. The reduced exposure to mechanical forces may allow for improved osseointegration resulting in greater extraction torques [26]. Repeated loading delays bone on-growth around the implant lessening osseointegration [1, 11, 26]. In this study, proximal screws exhibited lower PRT which was most likely due to higher absorption of repeated load than the distal screws. This phenomenon was less clearly observed in the ST screw group. This may be related to the already proven increased insertional torque and primary stability of the ST screws [9, 27–30]. PRT has been shown to have a positive correlation to the surrounding bone quality [3, 6, 11]. Several studies have shown that the tibiae have lower BMD in the proximal-mid part of the bone and greater in the distal portion [31–33]. The goats used for our study were adult, healthy, and free of lameness or pathologic bone condition. Thus, we would expect that BMD likely influenced some of the PRT results.

ST screws exhibited greater peak reverse torques (PRT) than NST screws after a period of 60 days of loading in a screw-plate-bone construct. The ST screw threads placed into the bone are expected to more closely contact the bone surface with compression as compared with NST screws due to the lack of the tapping process prior to the screw placement [1, 34]. The tap device designed for use with NST screws has been shown to have longer threads than the screws and this discrepancy creates a micro space between the screw thread and cut bone [34]. This incongruity can result in implant micromotion [34] which can reduce the primary stability of the screws. Several studies have shown that the ST screws exhibit greater peak insertional torque (PIT) than the NST screws [1, 5, 16, 35]. According to these studies, ST screws obtain greater primary stability than the NST screws [3] and show better interfacial stiffness at the implant-bone interface [4]. Micromotion causes filling of the space between the bone and the implant with fibrous tissue or encapsulation of the implant [5]. Moreover, this process can lead to excessive bone resorption and inflammation around the implant (peri-implantitis) [4, 5, 34]. These processes will result in reduced implant secondary stability which will negatively influence the longevity of the implant as reflected by decreased PRT. In contrast, the ST screws due to their greater insertional stress have been associated with increased incidence of bone damage promoting bone failure [1] and transcortical diaphyseal tibial fractures [34]. These incidences may reduce primary as well as secondary stability. In contrast, the number of transcortical diaphyseal tibial fractures in our study was greater within the NST screws than in ST screws.

The length of the NST and ST screws ranged between 18 and 24 mm in our study and all of the screws were placed bicortical. Previous research on a different length of the orthodontic implants (1.4–3.8 mm) did not show any significant correlation between the length of the implant and PRT as long as the implant was longer than 1.4 mm, which was considered as implants’ minimal length [36]. The minimal length of the cortical screw is considered when at least 3 threads of the implant can be placed through the far cortex in order to achieve the rigid fixation [1]. In this study in all cases at least 3 threads of the screw were anchored in the far cortex.

The mean PRT of ST and NST screws in this study are comparable with the previous studies on PRT of screw implants. PRT values vary between studies due to factors affecting the osseointegration process and different materials used for the biomechanical tests [3, 14, 37].

Reverse torque can be a valid method to assess the biomechanical properties of orthodontic implants. This method has been used to reach a better understanding of the osseointegration process [3–7, 10–16]. The term ‘integration strength’ refers to the force required to break the bond between the implant and the bone, and this can be measured with the PRT [4]. Okazaki et al. showed that insertional torque positively influenced PRT immediately after implant placement. However, the PRT decreased with healing time and showed no difference between the screws at weeks 6, 9, and 12 after insertion [4]. Biomechanical interlocking decreases over time but may increase again as remodeling of the surrounding bone takes place [5]. Histological examination of the bone healing process around titanium implants has shown that the existing bone initially resorbs at the bone-implant surface and is replaced by newly formed bone [5]. The screws in our study were used in a non-load sharing segmental defect of the tibiae model in goats for approximately 8 weeks resulting in varied reverse torques between ST and NST screws. Some investigators have observed a positive correlation between the bone-implant contact (BIC) and PRT [13, 15] while others claimed that the bone quality formed around the implant is more important than the amount [3, 6, 11, 14].

The main limitation of this study is the lack of measured peak insertional torque (PIT) during the screw placement. Even though the PIT defines implants primary stability [1, 26, 38] not their secondary stability [3] which was measured in this study, it could have been used to standardize the screw placement within the plate. In this study however, limitations of equipment and study design prevented measurement of insertion torque, therefore the variability of insertion torque may have contributed to differences in PRT. Next, the torque unit was limited in a range which resulted in the inability to measure low and high range torques. The torque cell had been selected based on expectations derived from previous studies. Finally, hence this in vivo study was a part of another research project, introducing the control group was not possible. The control group would have consisted of the screws on which the primary stability would have been measured. The screws would have been placed in the same fashion as described above and they would have been removed immediately after their placement. The PRT would have been measured right before the implant removal. These are the weaknesses that may be addressed in future work.

## Conclusion

The DCP-screw-bone construct is an adequate fixation method providing a sufficient stabilization in this 2.5 cm tibial defect model. The construct stabilization can be assessed by measuring implants osseointegration. The ST screws were shown to have a stronger bone-implant interface based on better PRT as compared with NST screws after 60 days post-implantation. Screws placed in the proximal tibia exhibited significantly lower peak reverse torque than those placed in the distal tibia. The lower reverse torque in the proximal tibia may be influenced by load distribution in the goats’ tibiae-plate assembly or because of different bone density between the proximal and distal parts of the bone. This phenomenon was less evident in the self-tapping screws presumably because of the greater primary stability as compared with non-self-tapping screws.

## Methods

**Fig. 1:**
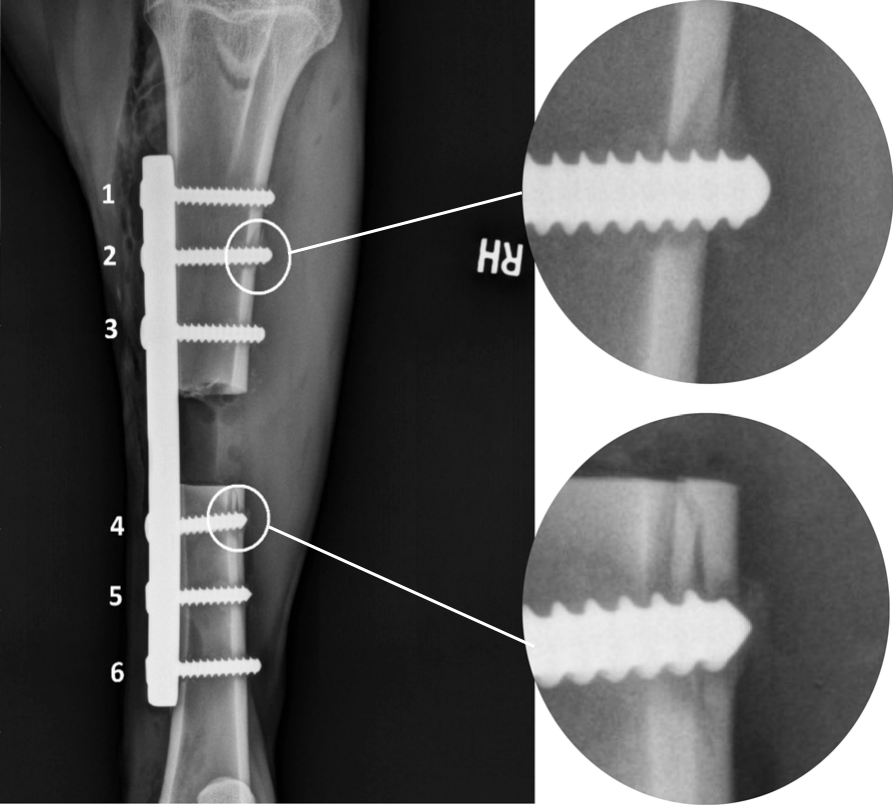
Goat tibial ostectomy model supported with an 8 – hole dynamic compression plate (DCP). The 2.5 cm defect was created in the mid-tibia and the plate was fixed with 6 ST or NST screws placed proximally to the ostectomy (pos. 1–3) and distally (pos. 4–6). The two white circles are labeling the transcortical diaphyseal tibial fractures

Animal study: The goats in the study were participating in a research project studying bone healing of a non-load sharing, mid-diaphysis segmental defect (2.5-cm length) of the tibia under an approved protocol (KSU IACUC # 2947) (Fig. 1). The animals participating in the study were mix bred adult (> 2 years old) female goats weighing 35 to 65 kgs purchased from the local vendors for the research purpose and owned by the university. The animals were healthy and without evidence of lameness or bone abnormalities. Briefly, the defect creation procedure was performed under general anesthesia which was maintained with the Isoflurane^1^ gas inhalant (2.5–4% MAC at the beginning of anesthesia and 1.5% MAC – 1.0% MAC during the procedure). The animals were sedated with 0.05 mg/kg, IV Xylazine^2^ (20 mg/ml) and induced with 5 mg/kg IV Ketamine^3^ (100 mg/ml) and 0.25 mg/kg IV Midazolam^4^ (5 mg/ml). During the defect creation procedure an 8 - hole 4.5 mm 316 L stainless steel DCP^5^ and 3.5 mm 316 L stainless steel cortical bone screws^5^ were used to stabilize the bone. Each bone segment (proximal, distal) received 3 screws. For statistical analysis, screw positions in the proximal bone segment were assigned positions 1, 2 and 3 from proximal to distal. Screws placed in the distal bone segment were assigned positions 4, 5 and 6 from proximal to distal. Goats were monitored for lameness daily during the study periods to assess the use of the operated limb.

In each goat, the DCP were fixed with only one type of screw, either NST cortical screws or ST cortical screws. All the screws used for this study were placed in standard AO/ASIF fashion and all were bi-cortical screws (near and far cortex). Briefly, the thread hole (2.4 mm diameter) was drilled with 12 V battery operated performance drill^6^ (maximum torque 19.21 Nm) and in the NST screws group it was tapped manually prior to the screw placement. Both screw types (NST and ST) were placed manually, using a handheld screwdriver. The screw lengths ranged between 18 mm to 24 mm, the core diameter equaled 2.4 mm, the thread diameter equaled 3.5 mm, and the thread pitch equaled 1.25 mm. The screws were inserted by three of the surgeons (DEA, JR, and JL) and the method was uniformly used by all surgeons. It has been recommended that the tapered tip and cutting flutes extend beyond the far cortex, therefore a care was taken that at least 3 threads of the screw were anchored in the far cortex to maintain rigid fixation [34]. All DC plates were fixed with 6 screws in total; 3 proximal to the osteotomy and 3 distal to the osteotomy (Fig. 1). The screws remained in place for 60 days [39] and were removed at the termination of the study period. Radiographic images were obtained for all goats on days 7, 14, 30, and 60 of the study periods, and they were evaluated to document the occurrence of transcortical diaphyseal tibial fractures in the cortices evident on radiographs and any change in screw-plate-bone interface, position, and fracture gap. After 60 days of the study period the animals were euthanized with overdose of pentobarbital administered intravenously. Euthanasia was induced by rapid intravenous injection into the jugular vein using pentobarbital (100 mg/kg body weight, IV) in accordance with the AVMA guidelines on the euthanasia of animals [40]. Pentobarbital rapidly induces unconsciousness without excitation. Death was confirmed by cessation of any detectable heartbeat and breathing, and loss of corneal reflexes. All implants were removed in the same manner, starting from the most proximal position [1] and following the order (from 1 to 6) until the most distal screw [6]. The plate was stabilized manually and therefore prevented from its movement during implant removal. The peak reverse torque for each screw was measured using a hand held torque driver.^7^ The pressure was applied gradually increasing, until the screw turned and then stopped. The torque driver did not require calibration and zeroing prior to the test. The hand held torque driver measured torque in the range between 0 and 22.6 Nm. After the implants had been removed and the tissues had been harvested for histopathology within the study on the bone regeneration, the cadavers were disposed at the Kansas State University.

Data was analyzed using a mixed-effects multinomial logistic regression model with the reverse torque categories as the multinomial outcome variable and the screw type (non-self-tapping and self-tapping) as well as screw position in the plate (proximal to distal with the increasing numbers from 1 to 6) as the fixed independent effects (multinomial exposure variable). The Odds Ratios, as well as 95% Confidence Intervals (95% CI) for fixed effects (screw type and screw position), were estimated with the reference to the screw position no. 6 and self-tapping screw type while holding other effects constant. Statistical significance was identified at the level of *p* < 0.05. The statistical analysis of the association between the transcortical diaphyseal tibial fractures and the screw type as well the fractures and PRT was done using two-sided Fisher’s exact test. Statistical Analysis was performed using PROC GLIMMIX in SAS9.4 TS1M4 for Windows 64x.^8^

## Data Availability

The datasets generated and/or analyzed during the current study are available in the DRYAD online repository, 10.5061/dryad.km78ms9

## Abbreviations

BIC: Bone Implant Contact
BMD: Bone Mineral Density
DCP: Dynamic Compression Plate
IV: Intravenous
MAC: Minimal Alveolar Concentration
NST: Non-self-tapping
PIT: Peak Insertional Torque
PRT: Peak Reverse Torque
RF: Resonance Frequency
ST: Self-tapping

## Footnotes

1 Isoflurane, Abbott Laboratories Inc., 100 Abbott Park Road, Abbott Park, IL 60064, USA

2 AnaSed, Lloyd Laboratories Inc., #10 Lloyd Avenue, First Bulacan Industrial City Brgy. Tikay, City of Malolos, Bulacan, Philippines

3 Ketamine, Pfizer Inc., 235 East 42nd Street NY, NY 10017, USA

4 Midazolam, AmerisourceBergen Inc., 1300 Morris Drive, Chesterbrook, PA 19087, USA

5 Dynamic Compression Plate, Self-tapping and Non-self-tapping screws, DePuy Synthes Inc., 325 Paramount Dr. Raynham, MA 02767, USA

6 Performax 12 V 3/8″ cordless drill, 5101 Menard Drive, Eau Claire, WI 54703, USA

7 Electrotorque System TQJE1500, Snap-on Inc., 2801 80th Streer Kenosha, WI 53143, USA

8 SAS Institute Inc. 100 SAS Campus Drive, Cary, NC 27513–2414, USA
